# Recent insights into the crosstalk between senescent cells and CD8 T lymphocytes

**DOI:** 10.1038/s41514-023-00105-5

**Published:** 2023-04-04

**Authors:** Ines Marin, Manuel Serrano, Federico Pietrocola

**Affiliations:** 1 Institute for Research in Biomedicine (IRB), Barcelona Institute of Science and Technology, 08028 Barcelona, Spain; 2 Cambridge Institute of Science, Altos Labs, Granta Park, Cambridge, CB21 6GP UK; 3 Department of Biosciences and Nutrition, Karolinska Institute, 14157 Huddinge, Sweden

**Keywords:** Cancer, Senescence

## Abstract

Recent reports in oncological and non-oncological experimental setups provide strong evidence that senescent cells are under the surveillance of CD8 T cell-mediated adaptive immunity. These new data also shed light on the mechanisms that sensitize senescent cells to CD8 T cell-dependent killing, as well as those that enable senescent cells to evade CD8 T cell immunosurveillance. Understanding the interplay between cellular senescence and the adaptive immune system may open new strategies to ameliorate aging and aging-associated diseases.

Cellular senescence is a non-apoptotic response to stress and damage that profoundly changes several aspects of normal cellular biology. In young, healthy, organisms, senescent cells are efficiently cleared by the immune system. However, upon aging or in immunodeficient or immunosuppressive contexts, senescent cells may accumulate and contribute to systemic aging and multiple pathologies^1^. Moreover, in response to cancer chemotherapy, senescent cells increase across the organism and intratumorally, where they can favor tumor progression by modifying the intratumoral microenvironment and interacting with the immune system^2^.

The alterations that characterize the process of cellular senescence equip senescent cells with the ability to engage in an extensive dialog with host leukocytes^3^^,4^. On the one hand, the secretion of soluble mediators by senescent cells (henceforth referred to as “senescence-associated secretory phenotype” or SASP) promotes the chemoattraction and activation of immune cells. The SASP is highly dynamic and context-dependent, instigating immune responses that can range from pro- to anti-inflammatory^3^. On the other hand, immune cells can influence the process of senescence, including the promotion of senescence and, its opposite, its clearance. There is compelling evidence supporting the concept that specific immune cells (e.g., neutrophils, CD4 T cells) promote the induction of senescence in normal and cancer cells both at the tissue and systemic levels^5–8^. In addition immune cells can eliminate senescent cells from tissues^9^. An ample body of literature links innate immune cells (e.g., NK cells, iNKT cells, macrophages, and neutrophils) to the elimination of senescent cells in both physiological and pathological circumstances^10–13^. Conversely, the signals that enable senescent cells to bypass the control of the innate immune system are also being unveiled, such as the upregulation of the non-canonical MHC-I molecule HLA-E^14^. Despite these important advances, the potential connections between senescent cells and the CD8 T cell arm of adaptive immunity have remained largely unexplored. A series of recent works contribute to shed new light on the multipronged interactions between senescent cells and CD8 T cells.

In two back-to-back articles published in *Cancer Discovery*, the laboratory of Scott Lowe and our laboratories provide mechanistic insights into the crosstalk between senescent cells and CD8 T cell-dependent adaptive immunity^15,16^. Both studies report that senescent cells are characterized by an elevated expression of MHC-I together with the molecular machinery required for the processing and presentation of antigens, rendering senescent cells highly sensitive to recognition and killing by CD8 T cells. Using various models of therapy-induced senescence in normal and neoplastic cells in vitro, Marin et al.^15^ found that MHC-I upregulation depends upon an intact paracrine type I interferon signaling, in thus far as IFN-I blockade or JAK inhibition blunts the effect. In addition, using primary human cancer cells and their autologous T-cell infiltrating lymphocytes (TILs), they demonstrate in ex vivo co-culture experiments that senescent cancer cells are superior in stimulating reactive TILs compared to their parental non-senescent cancer cells^15^. In Chen et al.^16^, they use a p53-deficient liver cancer model that undergoes senescence upon restoration of p53 using genetic tools. Interestingly, the induction of senescence in this in vivo model promotes the expression of IFNGR1. In turn, this enhances the capacity of senescent cells to respond to the IFN-γ produced by tumor-associated macrophages recruited in a SASP-dependent manner. The activation of IFN signaling is propaedeutic for MHC-I overexpression and subsequent CD8 T cell mediated killing^16^. These results are further supported by a recent preprint by Di Micco and colleagues who also report high levels of MHC-I in chemotherapy-treated primary human senescent leukemia cells^17^. Also, these findings are aligned with previous data from the Van Deursen and Schmitt labs placing macrophages at the interface between stressed/senescent cells and CD8 T cell mediated clearance^18,19^.

The identification of self-peptides enriched in senescent cells and able to trigger adaptive immunity (“seno-antigens”^20^) is an attractive possibility towards vaccines against senescent cells. In this context, pioneer work by Andrei Gudkov and colleagues observed that senescent cells can trigger the formation of antibodies and identified an oxidized form of vimentin as one of the recognized antigens present in senescent cells^21^. More recently, the group of Tohru Minamino reported that it is possible to trigger adaptive immunity—both mediated by B cells and by CD8 T cell—against a surface protein enriched in senescent cells, GPNMB (glycoprotein nonmetastatic melanoma protein B)^20^. Interestingly this type of vaccination against a “seno-antigen” reduced the burden of senescent cells in vivo, ameliorated the pathological effects of obesity and atherosclerosis, and extended the longevity of progeric mice^20^. The recent work by Marin and colleagues^15^ expands this concept by reporting that senescent primary fibroblasts exhibit a partially altered immunopeptidome, sufficient to evoke an antigen-specific CD8 T cell-dependent response when mice are immunized with senescent cells. Notably, a fraction of the senescence-associated antigens are not expressed by the parental non-senescent fibroblasts, further reinforcing the existence of senescence-specific MHC-I-associated peptides or “seno-antigens”. Following the advent of novel tools to decipher the immunopeptidome in vivo^22^ and characterize T cell receptors even with spatial resolution^23,24^, these observations open exciting avenues towards the identification of “seno-antigens” that can be therapeutically harnessed, for instance in the form of dendritic cell (DC)-based or mRNA vaccines, for educating the adaptive immune system to eliminate pathologic senescent cells in setups of aging or age-related diseases.

Besides directly boosting the immunogenicity of target cells, Marin and colleagues^15^ report that the induction of senescence in neoplastic cells also enhances CD8 T cell-dependent surveillance by promoting the recruitment and maturation of DCs. This effect is achieved via the release of adjuvant signals and the direct transfer of antigens from senescent cells to DCs to elicit the DC-dependent activation of T cells. In analogy to this finding, the use of senescent cells as vaccination agents prevents tumor formation in mice^15^. Indeed, senescent cancer cells produced a stronger immunization than cancer cells undergoing immunogenic cell death (ICD), which is the current gold standard for cancer vaccination. These observations are aligned with pioneering work from Ralph Weichselbaum and colleagues using senescent cells as cancer vaccination agents^25^. Additional data needed to elucidate the modalities of antigen transfer from senescent cells to DCs and the relevance of live senescent vaccines in non-oncological settings.

The experimental evidences presented above demonstrate potent immunostimulatory actions mediated by senescent cells. However, senescent cells accumulate in tissues during aging and multiple age-related pathologies indicating that senescent cells can somehow bypass CD8 T cell-mediated surveillance. While this effect can be partially due to the decline in the fitness of T lymphocytes during aging, an event that may temporally precede the accumulation of senescent cells in tissues, additional mechanisms are surfacing. Recent works by Makoto Nakanishi and Zhixun Dou groups suggest that non-neoplastic senescent cells can directly suppress the cytotoxic function of CD8 T cells by expression of PD-L1^26,27^. Both teams report that PD-L1 is overexpressed on the surface of senescent cells, and it is upregulated in aged and pathological tissues, including liver steatohepatitis and pulmonary fibrosis^26,27^. In line with this finding, long-term administration of anti-PD-1 monoclonal antibodies enhances CD8 T cell-dependent clearance of p16+ senescent cells and alleviates the pathological sequelae linked to the overabundance of senescent cells in contexts of natural aging and steatohepatitis^27^. These important findings are in line with analogous evidence in the context of cancer indicating that therapy-induced senescence results in the upregulation of PD-L1, PD-L2 and CD80^18,28,29^. Indeed, anti-PD-L2 treatment is strongly synergic in combination with standard senescence-inducing chemotherapy in mice, acting as a senolytic immunotherapy^27^. It is tempting to speculate that the overexpression of immune checkpoints signals by senescent cells may represent an adaptive response to prevent autoimmunity triggered by senescent cells. In the case of cancer cells, the same mechanisms may allow therapy-induced cancer senescent cells to evade immune clearance.

Taken together, these results add an additional layer of complexity to the multifarious processes through which senescent cells accumulate in tissues during aging. As postulated on the basis of heterochronic parabiosis experiments and models of tissue damage executed in young *versus* aged animals^30–32^, the sole time-dependent accumulation of ‘damage’ appears insufficient to explain the elevated burden of senescent cells in the aged organism. Instead, these data advocate for a composite model that considers enhanced rate of senescent cell formation, defective clearance, and increased resistance to elimination, all acting together as critical determinants of the accrued level of senescent across lifespan (Fig. 1).

**Fig. 1:**
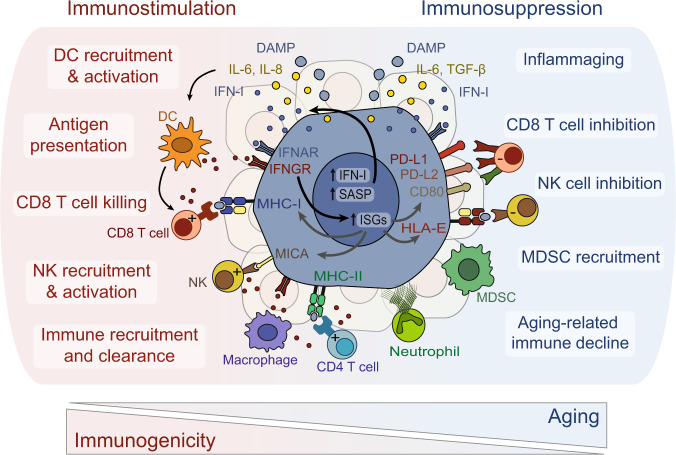
Immunomodulatory features of senescent cells. Senescent cells engage in an extensive dialog with host leukocytes. Depending on the physiopathological context, senescent cells display immunostimulatory, which pave the way for their effective clearance, or immunosuppressive attributes, which at least in part account for their accumulation and persistence in vivo. CD80 cluster of differentiation 80, DAMP damage associated molecular patterns, DC dendritic cell, IFN-I type I interferon, IFNAR interferon-α/β receptor, HLA-E HLA class I histocompatibility antigen, alpha chain E, IFNGR interferon-γ receptor, IL-6 interleukin 6, IL-8 interleukin 8, ISGs interferon-stimulated genes, MDSC myeloid derived suppressor cell, MICA MHC class I polypeptide–related sequence A, MHC-I major histocompatibility complex I, MHC-II major histocompatibility complex II, NK natural killer, PD-L1 programmed death-ligand 1, PD-L2 programmed death-ligand 2, SASP senescence-associated secretory phenotype, TGF-β transforming growth factor beta.

At the present stage of the literature, different modalities of immunotherapy have shown preclinical value in promoting the immune system-dependent clearance of senescent cells in contexts of natural/premature aging and various age-related diseases. These encompass CAR-T cells^33^ and antibodies^34^ to target surface proteins enriched in senescent cells, senolytic vaccination based on the immunization against “seno-antigens”^20^, and the use of immune checkpoint blockade as senolytic immunotherapy^27,28^. The results discussed here expand the toolbox of antisenescence immunotherapies to approaches that selectively activate T lymphocytes against senescent cells, such as DC-based vaccines. In turn, it appears conceivable to speculate that these interventions may also be combined to elicit a more durable immunological memory against pathologic senescent cell variants.
